# Applications of natural language processing tools in the surgical journey

**DOI:** 10.3389/fsurg.2024.1403540

**Published:** 2024-05-17

**Authors:** Khang Duy Ricky Le, Samuel Boon Ping Tay, Kay Tai Choy, Johan Verjans, Nicola Sasanelli, Joseph C. H. Kong

**Affiliations:** ^1^ Department of General Surgical Specialties, The Royal Melbourne Hospital, Melbourne, VIC, Australia; ^2^ Department of Surgical Oncology, Peter MacCallum Cancer Centre, Melbourne, VIC, Australia; ^3^ Geelong Clinical School, Deakin University, Geelong, VIC, Australia; ^4^ Department of Medical Education, The University of Melbourne, Melbourne, VIC, Australia; ^5^ Department of Anaesthesia and Pain Medicine, Eastern Health, Box Hill, VIC, Australia; ^6^ Department of Surgery, Austin Health, Melbourne, VIC, Australia; ^7^ Australian Institute for Machine Learning (AIML), University of Adelaide, Adelaide, SA, Australia; ^8^ Lifelong Health Theme (Platform AI), South Australian Health and Medical Research Institute, Adelaide, SA, Australia; ^9^ Division of Information Technology, Engineering and the Environment, University of South Australia, Adelaide, SA, Australia; ^10^ Department of Operations (Strategic and International Partnerships), SmartSAT Cooperative Research Centre, Adelaide, SA, Australia; ^11^ Agora High Tech, Adelaide, SA, Australia; ^12^ Monash University Department of Surgery, Alfred Hospital, Melbourne, VIC, Australia; ^13^ Department of Colorectal Surgery, Alfred Hospital, Melbourne, VIC, Australia; ^14^ Sir Peter MacCallum Department of Oncology, The University of Melbourne, Melbourne, VIC, Australia

**Keywords:** natural language processing, artificial intelligence, large language models, ChatGPT, generative pre-training transformer, surgery, surgical research, surgical education

## Abstract

**Background:**

Natural language processing tools are becoming increasingly adopted in multiple industries worldwide. They have shown promising results however their use in the field of surgery is under-recognised. Many trials have assessed these benefits in small settings with promising results before large scale adoption can be considered in surgery. This study aims to review the current research and insights into the potential for implementation of natural language processing tools into surgery.

**Methods:**

A narrative review was conducted following a computer-assisted literature search on Medline, EMBASE and Google Scholar databases. Papers related to natural language processing tools and consideration into their use for surgery were considered.

**Results:**

Current applications of natural language processing tools within surgery are limited. From the literature, there is evidence of potential improvement in surgical capability and service delivery, such as through the use of these technologies to streamline processes including surgical triaging, data collection and auditing, surgical communication and documentation. Additionally, there is potential to extend these capabilities to surgical academia to improve processes in surgical research and allow innovation in the development of educational resources. Despite these outcomes, the evidence to support these findings are challenged by small sample sizes with limited applicability to broader settings.

**Conclusion:**

With the increasing adoption of natural language processing technology, such as in popular forms like ChatGPT, there has been increasing research in the use of these tools within surgery to improve surgical workflow and efficiency. This review highlights multifaceted applications of natural language processing within surgery, albeit with clear limitations due to the infancy of the infrastructure available to leverage these technologies. There remains room for more rigorous research into broader capability of natural language processing technology within the field of surgery and the need for cross-sectoral collaboration to understand the ways in which these algorithms can best be integrated.

## Introduction

Natural language processing (NLP) is a subfield of artificial intelligence (AI) designed to use large language models to mimic human language processing abilities (1). NLP algorithms and technologies aim to receive, rationalise, interpret, and generate human language. The advent of widely accessible forms of this technology, including popular chatbot iterations and open-source foundation models, have led to global interest into the implementation of NLP into multiple sectors including healthcare (2).

The widespread digitalisation of healthcare, such as with electronic medical records, robotic surgery and telehealth, delivers significant potential for the implementation of NLP technology in surgery (3). To date, there is no widely implemented use of tools like ChatGPT in the surgical context. However, with the current digital infrastructure of healthcare and surgery, there is exciting potential for broad application of NLP for data analysis, risk prediction and prognostication, surgical communication, research and education (3). A greater understanding of these technologies, particularly for surgeons, healthcare providers and policy makers is fundamental in identifying best-practice methods in which these tools can be leveraged to improve the processes within surgery.

This review herein begins by providing a brief overview of the history and evolution of natural language processing technology and explores contemporary insights into the implementation of NLP technology throughout the journey of surgery.

## An overview of natural language processing

### History and evolution of natural language processing

Natural language processing draws its beginnings in the 1950s, where data scientists explored the use of machines to translate text between languages using rule-based approaches (4, 5). At this time, information retrieval was a separate entity to NLP, the latter solely focused on language output. These machine translation models utilised strict handwritten rules to translate text were significantly restrictive and could not translate contextual meaning within words (5). From the late 1960s, machine translator models virtually became non-existent. This led to the advent of lexical-analyser and parser generator tools which could transform text into smaller “tokens” and validate the token sequences respectively to understand and analyse human language (5). Despite this, these tools were limited by the handcrafted rules which defined them, leading to issues with interpreting “natural” spoken language including challenges with multiple interpretations of the same word or word sequence (5).

The 1980s saw the development of statistical NLP, which combined the rule-based methodology to statistical probability improvement in information gathering and improved natural language interpretation in a context-dependent manner (5). Significantly, the larger the amount of data used, the better these models were at these tasks, leading to these tools ability to deal with themes and emphasis in language (4). This paved the way for significant technological advancement in ths 1980s and 1990s, where sentence processing technologies were developed that could address more higher-level discourse and produce linguistically coherent and effective text for communication (4). These tools also demonstrated a sound ability to extract information and summarise it in an automated fashion (4).

The momentum in technological advancement continued in the 2000s, building on statistical NLP methodology. This began with neural language modelling (NLM) tools which were able to determine the probability of subsequent words in a sequence based on prior words. These technologies, through multiple cycles of progression led to the development of neural networks for NLP, which allowed neural network models at the time to process large amounts of text data and to understand complex patterns of language, enabling a myriad of linguistic tasks to be performed such as machine translation, thematic and sentiment analysis, information retrieval as well as text classification and summarisation (4). The neural networks however were limited by the long-term learning. Applications of deep learning technology to neural networks with novel transformer neural architecture saw the rise of newer models with longer-range learning capability. These models could be trained with massive amount of text data to predict subsequent words in text and were able to be fine-tuned to specific tasks at hand (4). Furthermore, they could generate natural text with contextual understanding (4). The ability of ongoing innovation in this space led to the development of large language models, powerful tools that had tremendous computational ability to analyse natural human language and generate natural language text (6). Popular and contemporary forms of these tools have included ChatGPT and Google Bard. At present, iterations of these technologies are numerous, with renowned large language models available on the market represented in Table 1.

**Table 1:** Examples of popular chatbot and open-source foundation large language models and associated developers of these models. In this representation, the term “foundation model” refers to large artificial intelligence models as defined by Stanford and the term “large language model” refers to language centred models.

Popular commercial large language models	• ChatGPT, GPT 3.5, GPT 4.0 (Open AI) • BARD (Google) • Bing Chat (Microsoft) • Jasper.ai (Jasper) • ChatSonic (Writesonic) • ERNIE (Baidu) • Copilot (Microsoft) • Amazon CodeWhisperer (Amazon) • YouChat (You.com)
Open-source foundation large language models	• LLaMA (Meta) • LLaMa 2 (Meta with Microsoft) • RedPajama (Together, Ontocord.ai, ETH DS3Lab, Stanford Centre for Research on Foundation Models, Hazy Research) • Flank-T5 (Google) • MPT (Mosaic ML) • BERT (Google) • LaMDA (Google) • ELECTRA (Google) • PEGASUS (Google) • BART (Meta) • RoBERTa (Meta) • MarianMT (Microsoft)

## Natural language processing and the journey of surgery

An overview of the studies included in the below discussion are available in Supplementary Table S1.

### Pre-operative applications

#### Triaging/referral process

The pre-operative period occupies a costly aspect of a patient's journey through surgery (7). Referrals are received through a myriad of sources including primary care, emergency and outpatient settings. NLP offers clinicians a potential way to automate this initial triaging process. Examples include a study performed Weissler et al.*,* where a NLP model was applied to 6,861 patients to identify clinically significant peripheral artery disease (8). The NLP model achieved a precision of 74% compared to the current algorithm approach which scored 65%. Similarly, Wissel et al. showed successful use of NLP to identify candidates for epilepsy surgery (9). Using a database of 519 epileptic patients, NLP was able to identify candidates who would require surgery with sensitivity of 0.80 and specificity of 0.77. Despite this, there is a potential for bias in this interpretation due to missing data from the retrospective records that were used for NLP training. In the more acute setting, a 2024 paper by Le et al. demonstrated the utility of several different Large Language Models (LLMs) in assessing fictional vascular surgery consultation queries to determine urgent need for surgical intervention (10). It was shown that while most of these models struggled with higher level decision making, they performed well with preliminary management suggestions with GPT 4 performing most reliably with a 100% accurate emergency identification (sensitivity and specificity of 100%) (10).

#### Surgical decision making

The ability of NLP to draw on pre-trained data suggest that when this data is linked with evidence-based databases, there is the potential for the development of a highly specific management tool. Perhaps in the future, NLP will sit alongside guidelines and treatment protocols to assist surgeons with treatment decisions. Preliminary evidence for this has already been seen with the use of ChatGPT. Haemmerli et al. demonstrated that ChatGPT was able to provide sound adjuvant treatment advice for glioma patients (11). ChatGPT also achieved moderate accuracy in identifying the most appropriate breast imaging procedure for patient screening and breast pain presentations (12). Furthermore, Cohen et al. demonstrated that NLP, when combined with machine learning, could predict candidates suitable for paediatric epileptic surgery with above average accuracy (*F*-values: 0.71–0.82) (13). Notably, ChatGPT and NLP in general lack the precision and dynamic decision-making capacity of qualified surgeons in these circumstances. Therefore, at this current point in time, it is unlikely these tools will autonomously make decisions for clinicians. However, as a supplement for clinicians, particularly those in areas with reduced access to resources such as medical literature subscriptions and guidelines, NLP algorithms may offer an openly accessible tool to assist surgeons with clinical decisions. This would need to occur after improved training and validation of these algorithms.

ChatGPT may assist surgeons to corroborate information required for the workup of surgical patients. This has been demonstrated through the use of NLP to assist with the diagnosis of inflammatory bowel disease (14). Specifically, the NLP algorithm was able to use a combination of progress notes, endoscopy, and pathology reports to correctly diagnose Crohn's disease (92%–98% positive predictive value) and ulcerative colitis (90%–97% positive predictive value) (15). Other uses of NLP that are similar include its role in identifying cancer phenotypes to assist clinicians with precision treatment, as well as in correlating mammographic and pathologic findings to assist surgeons with decision making (16, 17). The algorithms developed in these cases show high translatability to other settings. However, these studies are from single-institutions, with datasets that are derived from retrospective records, many of which are poorly characterised with respect to missing data and therefore the accuracy of their results is questionable. Larger studies are required to improve the evidence to allow for the generalisability and scalability of these tools.

Other potential efforts at mobilising NLP to assist surgeons have been centred around prognostication. Hu et al. created an NLP approach that was able to predict progression of glaucoma requiring surgery from clinical notes for patients with an area under the receiver operating characteristic of 73.4% (18). Parreco et al. utilised another NLP model to predict surgical ICU mortality using progress notes and severity scores with an AUC of 0.88 and accuracy of 94.6% (19). Other variations of this function include the ability predict length of hospital stay, readmission and discharge disposition, all with high accuracy (20–22).

#### Risk assessment

Another challenging facet of the pre-operative period is identifying patient risk factors to optimise prior to surgery. NLP shows promise in enhancing our ability to assess these risks. Suh et al. conducted a small volume study of 93 patients attending a pre-anaesthetic review (3). In their study, NLP software was used to identify relevant pre-operative history within clinical notes, which were the compared against notes made by anaesthetists. The NLP pipeline was able to identify relevant medical conditions that may present a risk to anaesthesia not noted by the anaesthetist in 16.57% of instances.

Moreover, the ability to identify specific medical conditions that pose a risk to surgery is an essential element of a pre-operative workup. Solomon et al. applied a NLP system to adult echocardiogram reports along with simple clinical data to identify clinically significant aortic stenosis (23). When compared against previously input diagnoses for aortic stenosis, the NLP system was able to achieve a much higher rate of accuracy. A staggering 927,884 echocardiograms were processed by the NLP system, which was able to classify 104,090 (11.2%) of the patients with aortic stenosis. This is in stark contrast to the 67,297 (64.6%) patients labelled with aortic stenosis originally. Importantly, amongst the 13.4% missed by manual coding, 19% had haemodynamically significant aortic stenosis. It is unclear whether the coding errors from this study translated to missed diagnoses and therefore inappropriate management of patients with clinically significant aortic stenosis, as this was not reported. Nonetheless, this highlights the value of NLP technology in improving pre-operative clinical coding and as an adjunct in peri-operative patient assessment to safely and efficiently risk stratify patients leading up to surgery. Importantly, the majority of studies were from single-institutions that utilised in-house NLP algorithms trained with retrospective medical record data. They subsequently evaluated these NLP technologies in an experimental environment composed of retrospective data of which missing data was poorly characterised. This therefore introduces bias in the ways the risks have been assessed with the need for more robust, prospective data to better inform the risk assessment potential of NLP technologies.

Parallel to the application of NLP in triaging surgical referrals, these systems may also be the solution to the inconsistent documentation of diagnoses (3). This inconsistency leads to inefficiencies during pre-operative workup including considerations into indications of surgery, risks of surgery and importantly, the patient's clarity of diagnosis (7). This is seen in above studies, with NLP systems outperforming current systems of diagnostic coding with aortic stenosis and peripheral artery disease (8, 23). Additionally, Li et al. looked at the potential application of NLP software to process magnetic resonance imaging (MRI) and knee arthroscopy reports to identify meniscal tears (24). Their software was able to identify disagreements between the knee MRI and arthroscopy reports with a sensitivity of 79% and specificity of 87%. Left unaddressed, these inconsistencies can lead to confusion for patients and non-surgical clinicians. Using NLP to reconcile these differences may help us avoid these complications in the patient's journey once the inherent biases in the current research are overcome.

### Intra-operative applications

The intra-operative use of NLP tools are perhaps the least considered applications of NLP in the field of surgery. However, the versatility of NLP offers potential to improve surgical efficiency within the operating room.

One area is during the process of generating operative notes. Operative notes are generally written in free-text form. This process, particularly after complex surgery, can be highly prone to errorincluding failure to capture all relevant parts of the procedure, or missing important detail (25). Electronic medical records, particularly with the ability to generate pre-filled checklists, are one solution to improving the quality of documentation (26). However, these proformas are limited by their ability to capture variations that occur, such as due to complex anatomy and development of intra-operative complications. NLP offers the potential for more real-time automated capturing of intra-operative to generate more accurate operative reports. Kunz et al. developed an NLP algorithm that could semi-automatically generate an operative report from a list of keywords for functional endoscopic sinus surgery that were dictated and recorded during the procedure, saving up to 30 min of time (27). While several limitations were identified before effective implementation of these processes could take place, including training of surgeons to relevant keywords for these models, the requirement of surgeons to wear microphones to dictate during the procedure, training of NLP algorithms to detect keywords vs. external dialogue and requirement for ongoing improvements in quality and accuracy of these generated operative notes, many solutions have been suggested (27). Theoretically, operative tools, such as the Da Vinci Robotic Assisted Systems, may be an avenue to explore with regard to integrating NLP technology due to presence of potentially compatible inbuilt microphones. Further developments into speech-to-text dictation software trained with NLP algorithms offer an exciting opportunity to improve speed, efficiency, accuracy of operative documentation of any surgical field.

The use of NLP in operative documentation could also be extended to coding and billing processes. Accurate coding and billing are important as demand for medical services increases to ensure appropriate management of overhead costs, appropriate funding of healthcare services and adequate remuneration of perioperative clinicians and surgeons. This process has been estimated to consume up to 10% of revenue (28). Attempts at automation have been stifled by challenges around hiring coding staff. A multicentre pilot study utilising a NLP algorithm for this purpose in spinal surgery demonstrated a near-human accuracy of 87% when compared with a senior billing coder, however was limited by a small dataset of keywords (29). Furthermore, NLP algorithms, when compared to current procedural terminology and international classification diseases (ICD) coding, has proven to more accurately identify intra-operative complications (30). These processes when combined with machine learning may also offer improved accuracy, however are limited by small datasets and potentially confounded by missing data (31). More robust research may offer the opportunity to develop, improve and extend NLP across surgical specialties, offering exciting potential for more accurate, efficacious and timely billing and coding to address inflationary pressures of healthcare resources (3).

The application of NLP in surgical decision making in the intra-operative period has been challenging.

A 2024 paper by Atkinson et al. proposed a use for NLP technology beyond assistance with documentation (32). In this study, researchers challenged ChatGPT-4 with six intra-operative, plastic surgery specific queries and assessed its qualitative accuracy of response through use of a purpose-built Likert scale (32). While the answers were generally described as accurate, the authors highlighted the issue of quality, with responses being closer to the level of a resident, as well as the familiar issue of accountability of decision making with the use of such tools. Other theoretical uses of NLP have been proposed in this space. One example is the integration of NLP technology into monitoring equipment to provide real-time warning messages in the event of deranged physiological parameters such as vital signs (33). Another example is the introduction of these technologies during surgical procedures to provide real-time feedback into the surgical steps taken, with the possibility to troubleshoot and gain advice when required to allow surgeons to make accurate decisions (33). These ideas however, remain in their infancy and lack the appropriate infrastructure at this point in time.

### Post-operative applications

#### Patient follow-up

Unique to modern NLP algorithms, such as ChatGPT has been the ability to generate meaningful responses to queries. This feature can be harnessed for provision of accessible information to patients. Recent implementation of ChatGPT for this purpose in the oral and maxillofacial surgery setting demonstrated that ChatGPT was able to answer common patient questions with no drop in quality, as judged by fully qualified surgeons of the same specialty (34, 35). Moreover, there has been theoretical consideration of utilising ChatGPT to provide personalized behavioural recommendations to patients following bariatric surgery, including recommendations for diet, physical activity and mental health (36). Similar to this, there is also suggestion that GPT-4 could provide personalised rehabilitation protocols based on current literature for the support of patients following joint arthroplasty (37). As these models are still in testing, the accuracy of information and advice provided must be taken with caution.

Similar findings were demonstrated in the ability of ChatGPT and ChatSonic in answering concerns from prostate cancer patients (38). Moreover, when applied to orthopaedics, ChatGPT demonstrated different answers when compared to a Google search of frequently asked questions related to hip and knee joint replacements (39). Furthermore, there is growing evidence that these technologies may be adapted to produce higher fidelity AI systems, such as natural-speech based algorithms to enhance follow-up (40). Despite these preliminary findings, the autonomous use of ChatGPT and other NLP algorithms by consumers without medical oversight poses potential harm to patients. Given the infancy of these tools and question about the accuracy of the medical data they provide, there must be quality assurance and safety mechanisms in place to ensure the information translated to patients is accurate, evidence-based and relevant to their medical context. However, these tools show promise in various processes of providing medical information including during the consent process, education about post-operative recovery and translating medical documentation into more digestible content to promote shared decision-making. Exercise must be cautioned if surgical patients are advised to use ChatGPT as a source of information, as would be provided if a patient were to gain information via Google search or social media.

#### Data extraction, audit and response to treatment

The digitalisation of healthcare records has led to the challenges of organising and working with big data. Manual extraction, collection and audit of such data is expensive, time-consuming and can introduce bias (41). Organised databases with defined parameters are one solution, with the American College of Surgeons National Surgical Quality Improvement Program being a notable example. This database has allowed surgeons to effectively audit, prognosticate and make evidence-based recommendations within their practice and research. NLP similarly offers a reliable and automated approach, through targeting individual keywords in multiple areas of interest to organise data in a more efficient manner.

NLP technologies are becoming more extensively trialled for this purpose in surgical oncology. Specifically, rule-based NLP tools have been shown to extract data from unstructured pathology reports with a high degree of accuracy. Abedian et al. demonstrated a NLP pipeline was able to identify four cancer subtypes (breast, prostate, colorectal, other) with 100% accuracy (42). Other studies have demonstrated outcomes ranging from 80%–100% indicating the accuracy of these tools is an area for ongoing optimisation and heavily dependent on the datasets and training algorithms applied (43, 44). Additionally, NLP has also demonstrated ability to extract additional key data in pathology reports, including parameters to allow for effective TNM staging of tumours and incomplete resection margins for cutaneous skin cancers, with high accuracy (42, 45, 46). Similar to this, NLP has also been tested in assessing response to treatment, with algorithms that examine pathology reports of breast cancer patients undergoing neoadjuvant therapy identifying complete pathological response with high sensitivity (90.5%) and accuracy (88.6%) (47).

NLP has also shown utility in extracting data from unstructured operative and radiology reports notes. Given the high volume of endoscopic procedures worldwide, NLP has been extensively trialled to extract data from colonoscopy reports with convincing outcomes. In particular, NLP has been demonstrated to extract key variables from colonoscopy reports including polyp characteristics (size, type, location) and bowel preparation quality with sensitivity and specificity of 95%–100% (48). Similar studies have confirmed accuracy levels ranging between 90%–100% for additional quality metrics including highest level of pathology, adenoma detection rate as well as other endoscopic modalities such as endoscopic retrograde cholangiopancreatography (ERCP) (49–51). Additionally, one multicentre study confirmed that when the performance of NLP was compared to that of gastroenterologists, the error rate was similar (49). For handwritten operative notes, the use of digital transfer technologies like optical character recognition tools allow NLP to operate at high levels of accuracy (52). NLP has also been applied to other surgical specialties with high accuracy, including in neurosurgery to identify incidental durotomy or intra-operative vascular injury and in orthopaedics to identify quality metrics in hip and knee arthroplasty (30, 53–55). When applied to radiology reports, NLP algorithms similarly achieve accuracy >90% through a variety of different applications. These include detection of intra-abdominal fluid suggestive of surgical site infection from CT reports, detection of bone metastasis from bone scintigraphy reports and characterisation of other surgical pathologies including periprosthetic fractures (56–58). Despite this, accuracy of NLP is highly variable, with one study identifying poor accuracy in identifying critical features on thyroid ultrasound including echogenicity (27%) and margins (58.9%) (59).

Perhaps the most widely studied application of NLP in data extraction has been from the medical record. One key area of interest is in the ability of using NLP technology for the identification of post-operative complications, with studies demonstrating performance similar to non-NLP and manual methods (60–62). Sohn et al. showed that when combined with machine learning, real-time identification of such outcomes could be achieved (62). Additional applications of NLP to the medical record have also been demonstrated, including the ability for surveillance of conditions such as for abdominal aortic aneurysms (AAA), development of clinical registries for rarer conditions such as intraductal mucinous pancreatic neoplasms (IPMN) and identifying palliative care benchmarks for surgery (63–65). To note, implementation of NLP technology did not occur autonomously and some form of oversight, such as with nurse supervision, was required (64).

Overall, it important to recognise NLP achieves high accuracy data extraction only within the purpose they have been specifically designed. This process is limited by the restricted training datasets that underpin the development of these tools.

## Academic applications

### Surgical education

NLP technology has shown undeniable potential for use in education, with many secondary and tertiary educators adopting these tools for curriculum and content development. There has also been widespread acknowledgement of the role of these tools in completing assessments and its potential impact on academic integrity (66, 67).

Recent studies have explored the use of ChatGPT and similar tools in surgical examinations. Ali et al. demonstrated that GPT-4, ChatGPT and BARD were able to complete the United States neurosurgical oral board preparatory examination questions, which assess higher-order diagnostic and therapeutic decision making in neurosurgery, with scores of 82.6%, 52.4% and 44.2% respectively (68). Hopkins et al. demonstrated ChatGPT achieved a result of 53.2% on neurosurgery board style questions (69). Similarly, across other surgical domains, Freedman et al. demonstrated GPT-4 was able to achieve 99th percentile in the 2022 Plastic Surgery In-service Training Examination assessing resident-level proficiency in plastic surgery and Oh et al. demonstrated GPT-4 achieved a result or 76.4% on the Korean General Surgery Board Examinations (70, 71). The variable outcomes achieved by these tools suggest NLP technology is not perfect. Rather, such tools rely on algorithms that utilise background training data to recognise statistical probability of words and therefore can match up strings of words that have correlation with each other in the specific context the tool is being applied (72). Therefore, accuracy of these tools reflects that of the training text and not from up-to-date surgical information that may be available. Alternatives such as BioGPT by Microsoft, a tool trained on PubMed literature, may offer a solution to providing evidence-based information. Considering the above, the ability for ChatGPT and its alternatives in producing pass results in higher-level surgical examinations suggest these tools may have potential in supporting the development of clinical decision making in surgical trainees. At the same time, these outcomes may also be a critique of current assessment and their superficial nature, highlighting the potential of NLP algorithms to be used in the development of more appropriate questions for higher level surgical assessment. NLP also offers candidates of such examinations another valuable tool for study, alongside modalities such as notes, flash cards, educational videos and presentations (73). For example, when comprehensive history and examination details are provided, NLP has the capacity to offer guidance on diagnostic and management options and may be a useful adjunct in supporting surgical education (71). Studies have supported this in the patient context, with ChatGPT demonstrated to comprehensively answer patient questions related to basic knowledge, lifestyle factors and treatment of cirrhosis and hepatocellular carcinoma (74).

NLP is also becoming increasingly adopted for use in the development of educational content. For training surgeons in the 21st century, many educators are utilising such tools for the delivery formal education based on current curriculum (66). The benefits of NLP for this purpose include opportunity to generate more creative approaches to lesson plans, identification of learning outcomes, development of new multiple-choice questions and ability to modify large bodies of texts, for example textbook chapters, into more contextualised content (75, 76). Implementation of similar principles to surgical education may offer more engaging ways of delivering surgical education, particularly related to concepts that are often difficult or time consuming to learn. However, the use of ChatGPT and similar tools should be exercised cautiously as there is risk of inaccurate information being presented. In an exploratory study assessing the ability of ChatGPT to develop a session of hyperlipidaemia, surgery was listed as a management modality and includes “LDL apheresis and bariatric surgery,” however, these modalities are not evidence-based standards for the management of isolated hyperlipidaemia, nor is LDL-apheresis a surgical option (72). The same study also showed ChatGPT failed to identify all important learning outcomes on hyperlipidaemia (72). Additionally, ChatGPT has been known to invent articles that have not existed (77). Surgical educators must consider an additional line of auditing teaching materials to distinguish between evidence-based real knowledge and convincingly written unverified information. For more formal educational content, such as delivery of assessable surgical lectures, there are many considerations prior to using NLP. Delivery of surgical education in tertiary institutions follows strict copyright guidelines and therefore there may be issues with compliance (75). Furthermore, there may be reduced engagement from surgical educators towards critical thinking and proactive development of contemporary teaching material if there is a shift towards reliance on these new technologies. This can lead to reduction in the quality of surgical educators and undesirable influences on aspects of teaching including bedside surgical teaching and intra-operative teaching. Additionally, ChatGPT and its alternatives currently lack the ability to generate images. Given the reliance on visual content in surgery, particularly for surgical anatomy and for operative teaching, the implementation of NLP tools should be used in conjunction with other resources including textbooks, scholarly articles and potentially AI image generators such as DALL-E2 by OpenAI.

Lastly, ChatGPT has shown promise in allowing educators to develop automatic grading and feedback of assessments to students. Furthermore, an NLP model has been shown to be able to classify quality of feedback that is provided to surgical residents prior to releasing such feedback (78). The use of such tools may allow training colleges mentors the opportunity to provide more quality feedback for ongoing clinical and professional development of surgical trainees.

### Surgical research

NLP offers an easily applicable method of extracting information from documents in a fraction of the time when compared to manual extraction (8, 11, 24, 79, 80). An important study by Xu et al. exhibited this utility of NLP technology in research, with 50 surgical reports put through an extended NLP system to facilitate automated coding (79). The system achieved precision of 95.4% when extracting features from pathology reports. Notably, the comparison was raised with manual coding, which would have taken an estimated 500 min against their extended NLP that only required 10 min. This highlights the tremendous ability of NLPs technology in the automated labelling and organisation of data from unstructured free-text. With the ever-evolving computerisation of health research, there comes an ever-increasing amount of electronic data, such as through large audit databases, published academic literature, electronic health records and online data storage. There is therefore need to explore new ways researchers can obtained and harness this data for research in an efficient manner. NLP may offer a solution to these challenges, including through its role in the automated labelling and categorisation of significant quantities of unstructured data.

## Ethical considerations and limitations

The ability of NLP to process human language and generate “thoughtful” responses provoke the question of whether we can be replaced by them in any capacity. In an interesting study by Zhu et al., five NLP systems were subject to 22 questions from a prostate cancer patient community (38). While the result was a surprising 90% across the five systems, the authors wrote about the obvious issues with our current technology, namely inability to ask further questions for clarification and inability to comfort patients. Haemmerli et al. assessed the abilities of ChatGPT by analysing 10 patients with primary CNS glioma and rating their recommendations between 0 and 10 with the help of seven CNS tumour experts (11). The outcome was poor performance in classifying glioma types with a median score of 3/10, albeit decent adjuvant treatment score with a median of 5/10. Important considerations outlined include suspicions of incorporated restrictions within ChatGPT with regards to generating medical advice, but also the suggestion that while lacking in nuance, ChatGPT and other NLP systems may serve as a useful adjunct to multidisciplinary decision workflow (11).

**Figure 1:**
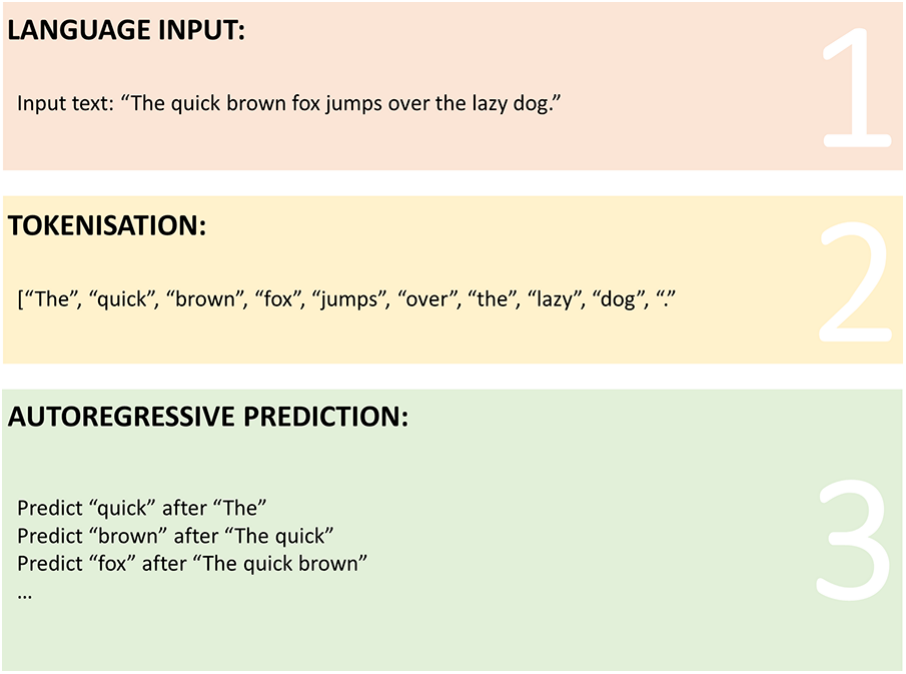
Graphical representation of tokenisation and autoregressive prediction processes in large language models.

In order for consumers of NLP technology to best understand the nuances in relation to the outputs from contemporary LLM algorithms, a foundational understanding of tokenisation and autoregressive function is required (81). The key steps that mediate this function are described in Figure 1. As a consumer of LLM and NLP technology, individuals provide input in the form of native language, such as a sentence or body of text (82). This text undergoes a process of “tokenisation” whereby it is broken into smaller units (tokens) which may be single words, sub-words or characters depending on the model; the benefit of which is to allow such models to process text efficiently and capture intricate linguistic patterns (82). Autoregressive function builds on from tokenisation by utilising previous training data to generate sequential prediction of text based on input data (82). Therefore, the quality of the training data is fundamental to the quality, content and realistic outputs from the model itself.

Given this understanding, a significant dangerous limitation to current NLP technologies is “hallucination” (82, 83). This concept was explored by Balas et al., in which ten ophthalmology patient cases were subjected to ChatGPT and Isabel NLP to assess for diagnosis (82). ChatGPT was able to provide a correct diagnosis in 9/10 cases, while Isabel provided only 1/10 provisional diagnosis correctly. The concept of hallucination was described in this study, whereby incorrect responses are generated with confidence, fooling the reader. More concerningly, this phenomenon appears to be more pronounced with highly technical content such as medical information as the processes of tokenisation and autoregressive encoding is highly influenced by their training datasets. Despite the ever-improving quality and complexity of contemporary NLP algorithms, hallucination renders these tools highly limited in surgery where strict control and regulation behind the type of information is warranted. Additionally, when it comes to the outputs of these algorithm, there is a degree of output volatility in instability of answers that are derived from these tools with consistent prompting. In particular, given the ways these tools are trained, there may be certain biases within these language models towards different answers based on the way consumers string their input entries (84). Specifically, the types of words placed near the end of the prompt can lead to specific patterns in outputs (84). This heterogeneity and volatility of outputs is a significant issue for healthcare service delivery, where there is a requirement for quality, evidence-based provision of information to an expected standard. Furthermore, surgeons must consider language alone is not sufficient in the delivery of quality surgical care. Linguistic patterns derived from these models may provide useful information when it comes to conceptualising complex ideas or significant amounts of data, however it is the surgeon's role to provide context, meaning and rationale to support these outputs to the diverse audience. A further contributor to this limitation is discussed in a review of ChatGPT within the healthcare sector by Li et al., whereby the privatisation of NLP systems may prevent enacting evidence-based changes in design (83). In their review, authors suggest withdrawing from product-based hype, and focusing research efforts to specialised language models designed for healthcare applications specifically, presenting a potential solution for this limitation.

The cost of implementing any new technology must be considered, and NLP is no exception. In their systematic review, Li et al. outlines the single-problem focus of ChatGPT, where accurate, high-quality information about one question cannot be generalised to all medical specialties (83). A downstream consequence of this includes the potential for a system where subspecialised NLP technology is developed for individual medical specialties which subsequently function in silos. In addition to being inefficient, the infrastructure and resources for development and implementation of these processes is anticipated to be financially costly and stakeholders involved in the distribution of resources must consider the practicality of these approaches. Further, a literature review of NLP in surgery identified additional costs with respect to time, such as in the tedious process of “cleaning” data for suitable use in a NLP algorithm (3). These sentiments were reflected in a study of patients who underwent breast biopsies. Buckley et al. used a NLP algorithm to convert unstructured reports to a machine-readable format and compared this against manual entry (85). The NLP software was able to identify 97% correct diagnoses, demonstrating an unacceptable margin of error in terms of pathology reporting. Moreover, contemporary NLP technology such as ChatGPT and BARD require tremendous computational capability made possible through the infrastructure and resources of the large corporations that developed them. It is likely in-house development of such technologies would not be feasible in the landscape of judicious healthcare funding and time pressures. Therefore, integration would rely on leveraging the capacities of these market leaders, which therefore raises the ethicolegal dilemmas of data stewardship, funding and confidentiality.

## Conclusion

This review presents a detailed exploration of the potential applications of natural language processing technologies, from pre-operative to post-operative stages of surgery, as well as in academia through applications in surgical education and research. At present, there is evidence to suggest these algorithms have the potential to outperform traditional manual tasks within surgery, including through the automation of triaging, data collection and audit, documentation and communication. This may lead to significant improvement in streamlining administrative and technical tasks within the field of surgery. However, the foundational literature behind the evidence is based on smaller, single-institution studies, highlighting the need for more rigorous research into broader applicability. Furthermore, there remains significant barriers to the widespread use of these technologies, including ethical considerations related to data stewardship, accuracy of information provided by language models and the cost of infrastructure to integrate these tools. More rigorous research into the applications of these technologies and further cross-sectoral collaboration is therefore required in order to efficaciously integrate natural language processing technology into the journey of surgery.
