# Supplementary material for: Applications of natural language processing tools in the surgical journey
Source: Front Surg. 2024 May 17;11:1403540. doi: 10.3389/fsurg.2024.1403540 (PMC11140056; doi:10.3389/fsurg.2024.1403540)
Supplement: Supplementary file 1 [file Table1.docx]

Supplementary Material

# Supplementary Data

Nil

# Supplementary Tables

Supplementary table 1. Overview of studies

| **Author** | **Year** | **Study design** | **Country of Publication** | **Number of institutions** | **NLP model** | **Training dataset** | **Clinical environment** |
| --- | --- | --- | --- | --- | --- | --- | --- |
| Abedian ~~(35)~~ (42) | 2021 | Non-randomised study | USA | 1 | Leo | Not mentioned | Analysis of retrospective data |
| Al-Afnan ~~(64)~~ (73) | 2023 | Empirical study | Kuwait | N/A | ChatGPT | - | No clinical data |
| Al-Haddad ~~(55)~~ (63) | 2010 | Non-randomised study | USA | 1 | In-house (REX) | Not mentioned | Analysis of retrospective data |
| Ali ~~(29)~~ (36) | 2023 | Letter to the editor | USA | N/A | GPT-4 | - | - |
| Ali ~~(37)~~ (44) | 2022 | Non-randomised study | UK | 1 | Retrospective clinical notes from 41 patients | Not mentioned | Analysis of retrospective data |
| Ali ~~(38)~~ (45) | 2023 | Non-randomised study | UK | Multiple | In-house | Not mentioned | Analysis of retrospective data |
| Ali ~~(59)~~ (68) | 2023 | Empirical study | USA | N/A | ChatGPT, GPT-4, Google Bard | - | No clinical data |
| Ananthakrishnan ~~(7)~~ (15) | 2013 | Non-randomised study | USA | 2 | In-house | Retrospective clinical notes from 1200 patients | Analysis of retrospective data |
| Atkinson (32) | 2024 | Qualitative observational study | Australia | N/A | ChatGPT | - | Theoretical clinical data |
| Baidoo-Anu ~~(68)~~ (77) | 2023 | Review | Canada | N/A | ChatGPT | - | - |
| Balas ~~(72)~~ (82) | 2023 | Non-randomised study | USA | 1 | ChatGPT, Isabel | - | Analysis of retrospective data |
| Balel ~~(31)~~ (34) | 2023 | Empirical study | Turkey | N/A | ChatGPT | - | No clinical data |
| Bian (40) | 2020 | Non-randomised study | China | 1 | In-house | Not mentioned | - |
| Boitano ~~(56)~~ (64) | 2023 | Non-randomised study | USA | 1 | In-house | Not mentioned | Analysis of prospective study period data |
| Bucher ~~(52)~~ (60) | 2020 | Non-randomised study | USA | 2 | In-house (EasyCIE) | Retrospective clinical notes from 4574 patients | Analysis of retrospective data |
| Buckley ~~(74)~~ (85) | 2012 | Non-randomised study | USA | 3 | In-house | Retrospective clinical notes from 1500 patients (500 from each institution) | Analysis of retrospective data |
| Chapman ~~(48)~~ (56) | 2017 | Non-randomised study | USA | 1 | In-house | Retrospective clinical notes from 249 patients | Analysis of retrospective data |
| Chen ~~(51)~~ (59) | 2020 | Non-randomised study | USA | 1 | In-house (cTAKES) | Not mentioned | Analysis of retrospective data |
| Cheng ~~(25)~~ (33) | 2023 | Letter to the editor | China | N/A | ChatGPT, GPT-4 | - | - |
| Cheng ~~(30)~~ (37) | 2023 | Letter to the editor | China | N/A | GPT-4 | - | - |
| Cohen ~~(28)~~ (13) | 2016 | Non-randomised study | USA | 1 | In-house | Retrospective clinical notes from 40-200 patients | Analysis of retrospective data |
| Danilov ~~(13)~~ (20) | 2022 | Non-randomised study | Russia | 1 | Ru-GPT3 | - | Analysis of retrospective data |
| Dubin ~~(33)~~ (39) | 2023 | Empirical study | USA | N/A | ChatGPT | - | No clinical data |
| Fevrier ~~(40)~~ (48) | 2020 | Retrospective cohort study | USA | 1 | In-house | Retrospective clinical notes from 1000 patients | Analysis of retrospective data |
| Freedman ~~(61)~~ (70) | 2023 | Empirical | USA | N/A | GPT-3.5,GPT-4 | - | No clinical data |
| Glaser ~~(39)~~ (46) | 2018 | Non-randomised study | USA | 1 | In-house | Retrospective clinical notes from 867 patients | Analysis of retrospective data |
| Groot ~~(49)~~ (57) | 2020 | Non-randomised study | USA, Netherlands | 2 | In-house | Retrospective clinical notes from 141 patients | Analysis of retrospective data |
| Haemmerli ~~(26)~~ (11) | 2023 | Non-randomised study | Switzerland | 1 | ChatGPT | - | Analysis of retrospective data |
| Han ~~(63)~~ (72) | 2023 | Empirical study | USA | N/A | ChatGPT | - | No clinical data |
| Hopkins ~~(60)~~ (69) | 2023 | Empirical study | USA | N/A | ChatGPT | - | No clinical data |
| Hou ~~(6)~~ (14) | 2014 | Review | USA | - | - | - | - |
| Hu ~~(11)~~ (18) | 2022 | Non-randomised study | USA | 1 | Bidirectional Encoder Representations from Transformers (BERT)-based models | Retrospective clinical notes from 3612 patients | Analysis of retrospective data |
| Imler ~~(41)~~ (49) | 2015 | Non-randomised study | USA | 1 | In-house | Retrospective clinical notes from 250 patients | Analysis of retrospective data |
| Imler ~~(43)~~ (51) | 2018 | Non-randomised study | USA | 1 | In-house | Retrospective clinical notes from 11387 patients | Analysis of retrospective data |
| ~~Jarvis (75)~~ | ~~2020~~ | ~~Review~~ | ~~USA~~ | ~~N/A~~ | ~~-~~ | ~~-~~ | ~~-~~ |
| Karhade ~~(14)~~ (21) | 2022 | Non-randomised study | USA | 2 | In-house | Retrospective clinical notes from 708 patients | Analysis of retrospective data |
| Karhade ~~(23)~~ (30) | 2021~~0~~ | Non-randomised study | USA | 5 | In-house | Retrospective clinical notes from 786 patients | Analysis of retrospective data |
| Karhade ~~(45)~~ (53) | 2022 | Non-randomised study | USA, Australia | 7 | In-house | Retrospective clinical notes from of patients (80% of total) | Analysis of retrospective data |
| Kasneci ~~(66)~~ (75) | 2023 | Position paper | Germany | N/A | ChatGPT | - | - |
| Khilnani ~~(67)~~ (76) | 2023 | Editorial | India | N/A | ChatGPT | - | No clinical data |
| Kim ~~(22)~~ (29) | ~~2022~~ 2023 | Retrospective cohort study | USA | 1 | In-house | Retrospective clinical notes from 391 patients | Analysis of retrospective data |
| Kim ~~(36)~~ (43) | 2014 | Non-randomised study | USA | 1 | In-house | Retrospective clinical notes from 100 patients | Analysis of retrospective data |
| Koleck ~~(34)~~ (41) | 2019 | Review | USA | N/A | - | - | - |
| Kooragayala ~~(71)~~ (80) | 2022 | Non-randomised study | USA | 1 | In-house | Retrospective clinical notes from 28 patients | Analysis of retrospective data |
| Kunz ~~(20)~~ (27) | 2022 | Proof-of-concept study | Germany | 1 | In-house | Retrospective clinical notes from 48 patients | Analysis of retrospective data |
| Laique ~~(44)~~ (52) | 2021 | Non-randomised study | USA | 2 | In-house (+optical character recognition) | Not mentioned | Analysis of retrospective data |
| Le (10) | 2024 | Qualitative observational stufy | USA | N/A | ChatGPT, Bard, Falcon 40B | - | Theoretical clinical data |
| Li ~~(17)~~ (24) | 2022 | Non-randomised study | USA | 1 | In-house | Retrospective clinical notes from 3593 patients | Analysis of retrospective data |
| Li ~~(73)~~ (83) | ~~2023~~  2024 | Review | Germany | N/A | ChatGPT | - | - |
| Lilley ~~(57)~~ (65) | 2018 | Perspective | USA | N/A | In-house | - | Analysis of retrospective data |
| Mohan (35) | 2023 | Qualitative descriptive study | USA | N/A | ChatGPT | - | Analysis of retrospective data |
| Morris ~~(2)~~ (3) | 2023 | Review | USA | N/A | ChatGPT | - | - |
| Muhlestein ~~(15)~~ (22) | 2021 | Non-randomised study | USA | 1 | In-house | Retrospective clinical notes from 595 patients | Analysis of retrospective data |
| Murff ~~(53)~~ (61) | 2011 | Non-randomised study | USA | 6 | In-house | Not mentioned | Analysis of retrospective data |
| Oh ~~(62)~~ (71) | 2023 | Empirical study | South Korea | N/A | ChatGPT, GPT-3.5, GPT-4 | - | No clinical data |
| Otles ~~(69)~~ (78) | 2021 | Non-randomised study | USA | N/A | In-house | Retrospective noted from 600 feedback comments | No clinical data |
| Parecco ~~(12)~~ (19) | 2018 | Non-randomised study | USA | 1 | In-house | Not mentioned | Analysis of retrospective data |
| Patel ~~(10)~~ (17) | 2016 | Non-randomised study | USA | 1 | In-house (MOTTE) | Not mentioned | Analysis of retrospective data |
| Rao ~~(27)~~ (12) | 2023 | Empirical study | USA | N/A | ChatGPT | - | No clinical data |
| Sagheb ~~(46)~~ (54) | 2021 | Non-randomised study | USA | 1 | In-house | Retrospective clinical notes from 420-1592 patients | Analysis of retrospective data |
| Savova ~~(9)~~ (16) | 2017 | Non-randomised study | USA | Not mentioned | DeepPhe | Not mentioned | Analysis of retrospective data |
| Sohn ~~(54)~~ (62) | 2017 | Non-randomised study | USA | 1 | In-house (NLP + machine learning) | Not mentioned | Analysis of retrospective data |
| Sok ~~(58)~~ (66) | 2021 | Review | Cambodia | N/A | ChatGPT | - | - |
| Solomon ~~(16)~~ (23) | 2021 | Non-randomised study | USA | 1 | In-house | Retrospective clinical notes from 1003 patients | Analysis of retrospective data |
| Suh ~~(3)~~ (7) | 2022 | Non-randomised study | USA | 1 | In-house | Retrospective clinical notes from 93 patients | Analysis of retrospective data from training set |
| Tibbo ~~(50)~~ (58) | 2019 | Non-randomised study | USA | 1 | In-house | Retrospective clinical notes from 1538 patients | Analysis of retrospective data |
| Tinmouth ~~(42)~~ (50) | 2023 | Non-randomised study | Canada | Ontario database (multi-institutional) | In-house | Retrospective clinical notes from 450 patients | Analysis of retrospective data from training set |
| Weissler ~~(4)~~ (8) | 2020 | Non-randomised study | USA | 1 | In-house ("PAD-ML") | Retrospective clinical notes from 6909 patients | Analysis of retrospective data |
| Wissel ~~(5)~~ (9) | 2020 | Non-randomised study | USA | 1 | In-house | Retrospective clinical notes from 4211 patients | Prospective analysis of live patients |
| Wu ~~(8)~~ (47) | 2023 | Retrospective cohort study | Canada | 4 | In-house | Retrospective clinical notes from 351 patients | Analysis of retrospective data |
| Wyles ~~(47)~~ (55) | 2022 | Non-randomised study | USA | 1 | In-house | Retrospective clinical notes from 519 patients | Analysis of retrospective data |
| Xu ~~(70)~~ (79) | 2004 | Non-randomised study | USA | 1 | MedLEE | Not mentioned | Analysis of retrospective data |
| Yeo ~~(65)~~ (74) | 2023 | Empirical study | USA | N/A | ChatGPT | - | No clinical data |
| Zaidat ~~(24)~~ (31) | 2023 | Non-randomised study | USA | 2 | XLNet | Retrospective clinical notes from 922 patients | Analysis of retrospective data |
| Zhu ~~(32)~~ (38) | 2023 | Letter to the editor | China | N/A | ChatGPT, YouChat, NeevaAU, Perplexity, Chatsonic | - | No clinical data |

## Supplementary Figures

Nil
